# Cosmetics-triggered percutaneous remote control of transgene expression in mice

**DOI:** 10.1093/nar/gkv326

**Published:** 2015-05-05

**Authors:** Hui Wang, Haifeng Ye, Mingqi Xie, Marie Daoud El-Baba, Martin Fussenegger

**Affiliations:** 1 Department of Biosystems Science and Engineering, ETH Zurich, Mattenstrasse 26, CH-4058 Basel, Switzerland; 2 Shanghai Key Laboratory of Regulatory Biology, Institute of Biomedical Sciences and School of Life Sciences, East China Normal University, Dongchuan Road 500, 200241 Shanghai, China; 3 Département Génie Biologique, Institut Universitaire de Technologie, F-69622 Villeurbanne Cedex, France; 4 Faculty of Science, University of Basel, CH-4058 Basel, Switzerland

## Abstract

Synthetic biology has significantly advanced the rational design of trigger-inducible gene switches that program cellular behavior in a reliable and predictable manner. Capitalizing on genetic componentry, including the repressor PmeR and its cognate operator O_PmeR_, that has evolved in *Pseudomonas syringae pathovar tomato* DC3000 to sense and resist plant-defence metabolites of the paraben class, we have designed a set of inducible and repressible mammalian transcription-control devices that could dose-dependently fine-tune transgene expression in mammalian cells and mice in response to paraben derivatives. With an over 60-years track record as licensed preservatives in the cosmetics industry, paraben derivatives have become a commonplace ingredient of most skin-care products including shower gels, cleansing toners and hand creams. As parabens can rapidly reach the bloodstream of mice following topical application, we used this feature to percutaneously program transgene expression of subcutaneous designer cell implants using off-the-shelf commercial paraben-containing skin-care cosmetics. The combination of non-invasive, transdermal and orthogonal trigger-inducible remote control of transgene expression may provide novel opportunities for dynamic interventions in future gene and cell-based therapies.

## INTRODUCTION

Synthetic trigger-controlled gene switches that enable spatio-temporal fine-tuning of transgene expression have been instrumental for functional genomic research (1), drug discovery (2) and the manufacturing of difficult-to-produce drug targets (3) and protein therapeutics (4). During the past decade synthetic biology, the science of reassembling cataloged and standardized biological items in a systematic, rational and predictable manner to create, engineer and program functional biological designer devices, systems and organisms with novel and useful functions (5–10) has significantly advanced the design of gene switches. They evolved from simple control devices providing trigger-inducible transgene expression (11–15) to complex transcription/translation networks enabling oscillating expression dynamics (16), intercellular communication (17) and fundamental arithmetic operations (18,19). Today, gene switches form the basis for the design of therapeutic gene networks that have been successfully validated in cell-based therapies using animal models of prominent human disorders (2,4,20–29).

Short-chain alkylated parabens are a group of plant antimicrobial defense metabolites (e.g. methylparaben (MP) is found in oca and grapefruit seeds, (30,31)) that have been clinically licensed by the FDA as well as approved within the European Union as food additives (E218, MP; E214, ethylparaben (EP); E216, propylparaben (PP); E209, heptylparaben) and have been widely used for over 60 years as preservatives in food, cosmetics and pharmaceuticals (32,33). Parabens (i) are inexpensive due to their simple high-volume industrial production, (ii) transdermally absorbed (34–36), (iii) rapidly reach the bloodstream (33,36), (iv) are rapidly metabolized and (v) renally cleared and (vi) are generally regarded as safe (37).

We have engineered paraben-repressible and -inducible transgene expression systems based on the genetic componentry of the Gram-negative bacterium *Pseudomonas syringae pathovar tomato* DC3000, a plant pathogen that causes bacterial specks of tomato (38). Expression of *P. syringae's* major multidrug efflux pump MexAB-OprM is regulated by PmeR (*Pseudomonas* multidrug efflux regulator), a TetR-type transcriptional repressor that binds to an inverted repeat (O_PmeR_) overlapping with the promoters driving *mexAB-oprM* and *pmeR* (39,40). Parabens have been shown to induce the expression of the *mexAB-oprM* genes by binding to PmeR and disrupting the PmeR–O_PmeR_ interaction, thereby conferring resistance to multiple plant defense metabolites including parabens (40,41). Taking advantage of the paraben-responsive PmeR–O_PmeR_ interaction, we have designed a set of mammalian gene switches that allow paraben-repressible as well as -inducible transgene expression in a variety of human cell lines. Furthermore, topical application of commercial paraben-containing skincare products was able to remote control transgene expression in subcutaneous (SC) designer cell implants in mice, suggesting that this technology will be compatible with future clinical applications.

## MATERIALS AND METHODS

### Plasmid design

Comprehensive design and construction details for all expression vectors are provided in Table 1. The assembly of some plasmids required annealing of complementary oligonucleotides: 50 pmol of each oligonucleotide was mixed in 50 μl ddH_2_O-diluted 1x NEB Buffer 4 (New England Biolabs, Ipswich, MA, USA), heated for 10 min at 95°C, cooled down over 4 h to 22°C and incubated at 22°C for another 2 h prior to cloning into the corresponding vector backbone. All relevant genetic components have been confirmed by sequencing (Microsynth, Balgach, Switzerland).

**Table 1.** Plasmids and oligonucleotides designed and used in this study

Plasmid	Description	Reference
pSEAP2-Control	Constitutive mammalian SEAP expression vector (P_SV40_-SEAP-pA).	Clontech, CA
pUC57	pUC19-derived prokaryotic expression vector	GeneScript, NJ
pZeoSV2(+)	Constitutive mammalian expression vector encoding the zeocin resistance gene (P_hCMV_-*zeo*-pA).	Invitrogen, CA
pMG11	Constitutive mammalian TtgA_1_ expression vector (P_SV40_-TtgA_1_-pA).	(12)
pSAM200	Constitutive mammalian tTA expression vector (P_SV40_-tTA-pA).	(14)
pWW124	γ-butyrolactone (SCB1)-repressible SEAP expression vector (P_SPA_-SEAP-pA).	(15)
pKR71	Constitutive mammalian KstR-KRAB expression vector (P_SV40_-KstR-KRAB-pA).	unpublished
pMM15	Mammalian expression vector (P_SV40_-pA).	unpublished
pMX101	Constitutive mammalian TtgS expression vector (P_SV40_-TtgS-pA; TtgS, TtgR-KRAB). TtgR was excised from pMG11 using *Not*I/*Bss*HII and ligated into the corresponding sites (*Not*I/*Bss*HII) of pWH9.	This work
pWH1	pUC57 containing a custom-designed mammalian codon-optimized PmeR	This work
pWH5	Paraben-inducible SEAP expression vector (P_PMS_-SEAP-pA; P_PMS_, P_SV40_-O_PmeR2_). Oligonucleotides OWH5: 5′-agcttGACGTC**ATACTTACATTCGCGGTTGTTTGTAAACATACTTACATTCGCGGTTGTTTGTAAAC**CCTGCAGGg-3′ and OWH6: 5′-aattcCCTGCAGG**GTTTACAAACAACCGCGAATGTAAGTATGTTTACAAACAACCGCGAATGTAAGTAT**GACGTCa-3′ were annealed and cloned into the *Hin*dIII/*Eco*RI-restricted pSEAP2-Control.	This work
pWH8	Constitutive PMA expression vector (P_SV40_-PMA-pA; PMA, PmeR-VP16). PmeR was PCR-amplified from pWH1 with oligonucleotides OWH11: 5′-atatttgcggccgcGCCACCATGGTCAGACG-3′ and OWH13: 5′tattggcgcgcGGCTGTACGCGGACAGGCGTTCTCTTTCCACGTT-3′, restricted with *Not*I/*Bss*HII and cloned into the corresponding sites (*Not*I/*Bss*HII) of pSAM200.	This work
pWH9	Constitutive PMS expression vector (P_SV40_-PMS-pA; PMS, PmeR-KRAB). PmeR was PCR-amplified from pWH1 with oligonucleotides OWH11: 5′-atatttgcggccgcGCCACCATGGTCAGACG-3′ and OWH13: 5′-tattggcgcgcGGCT GTACGCGGACAGGCGTTCTCTTTCCACGTT-3′, restricted with *Not*I/*BssH*II and cloned into the corresponding sites (*Not*I/*Bss*HII) of pKR71.	This work
pWH10	Paraben-repressible SEAP expression vector (P_PMA_-SEAP-pA; P_PMA_, O_PmeR2_- P_hCMVmin_). O_PmeR2_ was excised from pWH5 using *Aat*II/*Sbf*I and ligated into the corresponding sites (*Aat*II/*Sbf*I) of pWW124.	This work
pWH19	Phloretin-inducible SEAP expression vector (P_TtgS_-SEAP-pA; P_TtgS_, P_SV40_-O_TtgR2_). Oligonucleotides OWH21: 5′- agcttGACGTC**CAGTATTTACAAACAACCATGAATGTAAGTATATTCCAGTATTTACAAACAACCATGAATGTAAGTATATTC**CCTGCAGGg -3′ and OWH22: 5′-aattcCCTGCAGG**GAATATACTTACATTCATGGTTGTTTGTAAATACTGGAATATACTTACATTCATGGTTGTTTGTAAATACTG**GACGTCa -3′ were annealed and cloned into the *Hin*dIII/*Eco*RI-restricted pSEAP2-Control.	This work

**Oligonucleotides:** Restriction endonuclease-specific sites are underlined, annealing base pairs are indicated in capital letters and the operator module O_PmeR_ is shown in bold. **Abbreviations**: **KRAB**, Krueppel-associated box protein of the human *kox-1* gene; **KstR**, *Mycobacterium tuberculosis* repressor of the cholesterol catabolism; **O_PapRI_**, SCA-specific operator; **O_PmeR2_**, tandem PmeR-specific operator; **O_TtgR2_**, tandem TtgR-specific operator; **pA**, polyadenylation site; **PmeR**, repressor of the *Pseudomonas syringae pathovar tomato* DC3000-drived multidrug efflux pump; **P_hCMV_**, human cytomegalovirus immediate early promoter; **P_hCMVmin_**, minimal version of P_hCMV_; **PMA**, PmeR-derived paraben-dependent transactivator (PmeR-VP16); **PMS**, PmeR-derived paraben-dependent transrepressor (PmeR-KRAB); **P_SPA_**, γ-butyrolactone (SCB1)-repressible promoter (O_PapRI_-P_hCMVmin_); **P_PMA_**, paraben-repressible promoter (O_PmeR2_-P_hCMVmin_); **P_PMS_**, paraben-inducible promoter (P_SV40_-O_PmeR2_); **P_SV40_**, simian virus 40 promoter; **P_TtgS_**, phloretin-inducible promoter (P_SV40_-O_TtgR2_); **SCA**, SCB1-dependent transactivator (ScbR-VP16); **SCB1**, *Streptomyces coelicolor* 1,2-(1'-hydroxy-6-methylheptyl)-3-(hydroxymethyl)-butanolide; **ScbR**, *Streptomyces coelicolor* γ-butyrolactone (SCB1)-specific quorum-sensing receptor; **SEAP**, human placental secreted alkaline phosphatase; **TetR**, *Escherichia coli Tn*10-derived tetracycline-dependent repressor of the tetracycline resistance gene; **tTA**, tetracycline-dependent transactivator (TetR-VP16); **TtgA_1_**, phloretin-dependent transactivator (TtgR-VP16); **TtgR**, repressor of the *Pseudomonas putida* DOT-T1E ABC multidrug efflux pump; **TtgS**, TtgR-derived phloretin-dependent transsilencer (TtgR-KRAB); **VP16**, *Herpes simplex* virus-derived transactivation domain; ***zeo***, zeocin resistance gene.

### Cell culture and transfection

Human embryonic kidney cells (HEK-293T, ATCC: CRL-11268), human cervical adenocarcinoma cells (HeLa, ATCC: CCL-2), human fibrosarcoma cells (HT-1080, ATCC: CCL-121), telomerase-immortalised human mesenchymal stem cells (hMSC-TERT, (42)) and baby hamster kidney cells (BHK-21, ATCC: CCL-10) were cultured in Dulbecco's modified Eagle's medium (Invitrogen, Basel, Switzerland; cat. no. 52100–39) supplemented with 10% (v/v) fetal bovine serum (FBS; Sigma-Aldrich, Buchs, Switzerland; cat. no. F7524, lot no. 022M3395) and 1% (v/v) penicillin/streptomycin solution (Biowest, Nuaillé, France; cat. no. L0022–100). Wild-type Chinese hamster ovary cells (CHO-K1, ATCC: CCL-61) were cultured in ChoMaster^®^ HTS (Cell Culture Technologies, Gravesano, Germany) supplemented with 5% (v/v) FBS and 1% (v/v) penicillin/streptomycin solution. All cell lines were cultured at 37°C in a humidified atmosphere containing 5% CO_2_.

All cell lines were transfected using an optimized polyethyleneimine (PEI)-based protocol (43). In brief, adherent cells cultivated in 24-well plates (50 000 cells in 500 μl medium per well) were incubated with 100 μl of a 1:3 PEI:DNA mixture (w/w) (PEI; MW 40 000, stock solution 1 mg/ml in ddH_2_O; Polysciences, Eppelheim, Germany; cat. no. 24765-2) containing 0.6 μg of total DNA. After 6 h, the culture medium was replaced by 500 μl PEI-free medium containing different concentrations of parabens. Cell concentration and viability were profiled with a CASY^®^ Cell Counter and Analyser System Model TT (Roche Diagnostics GmbH, Mannheim, Germany).

### Construction and characterization of stable cell lines

The HEK-PAR_OFF_ cell line, transgenic for paraben-repressible secreted alkaline phosphatase (SEAP) expression, was constructed by co-transfection of HEK-293 cells with a 10:5:1 (w/w/w) mixture of pWH8 (P_SV40_-PMA-pA), pWH10 (P_PMA_-SEAP-pA) and pZeoSV2(+) (P_SV40_-*zeo*-pA), followed by selection in culture medium containing 1 mg/ml zeocin (Invitrogen, cat. no. R250-05) and FACS-mediated single-cell cloning. Six out of 30 cell clones were randomly picked and the best-in-class HEK-PAR_OFF1_ was used for all follow-up studies. Likewise, the HEK-PAR_ON_ cell line, transgenic for paraben-inducible SEAP expression, was constructed by co-transfection of HEK-293 cells with an 8:8:1 (w/w/w) mixture of pWH9 (P_SV40_-PMS-pA), pWH5 (P_PMS_-SEAP-pA) and pZeoSV2(+) (P_SV40_-*zeo*-pA) followed by selection in culture medium containing 1 mg/ml zeocin and FACS-mediated single-cell cloning. Six out of 30 cell clones were randomly chosen and the best-in-class HEK-PAR_ON6_ cell line was used for all follow-up studies.

### Quantification of reporter protein production

Production of human placental SEAP was quantified in the culture supernatant by measuring the colorimetric absorbance time course of the SEAP-mediated *p*-nitrophenylphosphate to *p*-nitrophenolate conversion, as described previously (44). In brief, 120 μl of buffered substrate solution (100 μl of 2x SEAP assay buffer [20 mM homoarginine, 1 mM MgCl_2_, 21% diethanolamine, pH9.8] and 20 μl substrate solution [120 mM *p*-nitrophenylphosphate]) was added to 80 μl heat-inactivated (65°C, 30 min) cell culture supernatant and the light absorbance time course was measured at 405 nm (37°C). The SEAP levels in the bloodstream were profiled using a chemiluminescence-based assay (Roche Diagnostics GmbH, Mannheim, Germany; cat. no. 11 779 842 001).

### Chemicals and cosmetics

Ethanol (EtOH; cat. no. 02860), dimethyl sulfoxide (DMSO; cat. no. D8418), MP (cat. no. H3647), EP (cat. no. 11988), PP (cat. no. P53357), butylparaben (BP; cat. no. 54680), isobutylparaben (iBP; cat. no. 715077) and phloretin (cat. no. P7912) were purchased from Sigma-Aldrich (Buchs, Switzerland). All parabens were prepared and stored as 20 mM stock solutions in 50% EtOH (in ddH_2_O). Phloretin was prepared and stored as a 50 mM stock solution in 100% EtOH. For animal experiments, different doses of PP were prepared in 100% DMSO before the treatment. Kamill^®^ hand cream (Kamill^®^ Hand & Nagelcreme Classic; Burnus, Darmstadt, Germany) was diluted 200x in 25% DMSO (in ddH_2_O). Cien^®^ shower gel (Cien^®^ spring bloom magic shower gel; Lidl Stiftung & Co. KG, Neckarsulm, Germany) and Dove^®^ shower gel (Dove^®^ Dusche Schuetzende Pflege; Unilever, Hamburg, Germany) were diluted 100x in 25% DMSO (in ddH_2_O). Lancaster^®^ cleanser (Lancaster^®^ Express Cleanser, Lancaster, Paris, France) and Lancôme Paris^®^ toner (Lancôme Paris^®^ softening hydrating toner, L'Oreal, Paris, France) were diluted 10x and 5x in ddH_2_O, respectively.

### Animal experiments

Designer cell implants were produced by microencapsulating pWH9/pWH5-transgenic HEK-293 into coherent alginate-poly-(L-lysine)-alginate beads (400 μm diameter; 200 cells/capsule) using an Inotech Encapsulator Research Unit IE 50R (Buechi Labortechnik AG, Flawil, Switzerland) set to the following parameters: 0.2 mm nozzle with a vibration frequency of 1025 Hz, 25-ml syringe operated at a flow rate of 410 units and 1.12 kV for bead dispersion (26). One hour after intraperitoneal (IP) or SC (lower dorsum) implantation of 1×10^4^ microcapsules into eight-week-old female OF1 mice (oncins France souche 1; Charles River Laboratories, France), the animals were treated with PP injections (0–10 mg/kg in 50 μl DMSO, once every 24 h) or topical application of commercial hand cream (Kamill^®^, 600 mg, 3x every 8 h) and cleanser (Lancaster^®^, 600 μl, 3x every 8 h) or solutions containing three different concentrations of PP (50 μl, 3x every 8 h for a total of 0, 10, 100 mg/kg day^−1^). Blood samples were collected 48 h after implantation and serum was isolated using microtainer serum separating tubes (SST) tubes according to the manufacturer's instructions (centrifugation for 5 min at 10 000x*g*; Becton Dickinson, Plymouth, UK; cat. no. 365967). Semi-quantitative analysis of blood-paraben levels was performed by injecting mice once with 300 mg/kg of PP, collecting blood samples after 24 h and adding 10 μl of serum to 2.5x 10^4^ pWH9/pWH5-transfected HEK-293 cells, before SEAP expression was profiled and compared to a standard curve after 48 h.

All experiments involving animals were performed according to the directive of the European Community Council (2010/63/EU), approved by the French Republic (no. 69266310; project no. DR2013-01 (v2)) and carried out by Marie Daoud-El-Baba at the Institut Universitaire de Technologie, IUT, F-69622 Villeurbanne Cedex, France.

## RESULTS

### Design, construction and validation of paraben-repressible and -inducible mammalian transgene expression switches

In *Pseudomonas syringae* pv. *tomato* DC3000, the repressor PmeR (*Pseudomonas* multidrug efflux regulator) is released from promoters containing specific O_PmeR_ operator sites upon interaction with plant defense metabolites of the paraben class, to induce the paraben-eliminating multidrug efflux pump MexAB-OprM and establish resistance to plant-derived antimicrobial compounds (39,40). Capitalizing on the paraben-sensitive PmeR–O_pmeR_ interaction, we have designed two isogenic synthetic mammalian gene switches that either repress (PAR_OFF_) or induce (PAR_ON_) transgene expression in response to FDA-approved parabens.

**Figure 1.**
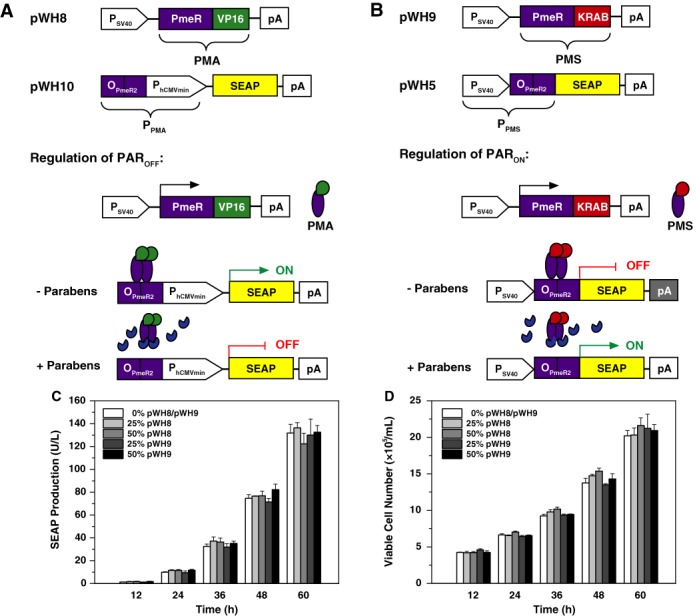

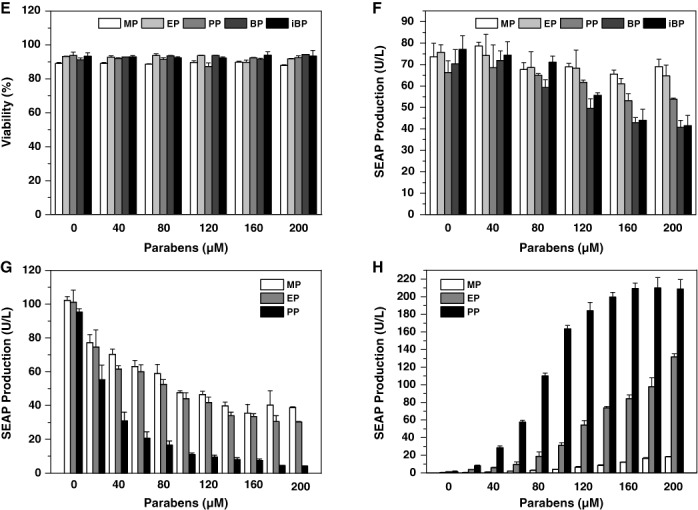
Design and validation of paraben-controlled mammalian transgene expression systems. **(A)** Design and functionality of the paraben-repressible transcription-control system PAR_OFF_. The synthetic mammalian paraben-mediated transactivator PMA (pWH8, P_SV40_-PMA-pA; PMA, PmeR-VP16) was designed by C-terminal fusion of PmeR (*Pseudomonas* multidrug efflux regulator) to the *Herpes simplex* virus-derived transactivation domain VP16. Following constitutive expression by P_SV40_, PMA binds and activates the chimeric promoter P_PMA_ (pWH10, P_PMA_-SEAP-pA; P_PMA_, O_PmeR2_-P_hCMVmin_) containing a tandem PMA-specific operator module O_PmeR2_ 5’ of P_hCMVmin_, which is set to drive expression of SEAP. In the absence of parabens PMA binds P_PMA_ and drives SEAP expression, while the presence of paraben derivatives results in the release of PMA from O_PmeR2_ and represses SEAP expression. **(B)** Design and functionality of a paraben-inducible transcription-control system PAR_ON_. The synthetic mammalian paraben-mediated transsilencer PMS (pWH9, P_SV40_-PMS-pA; PMS, PmeR-KRAB) was designed by C-terminal fusion of PmeR to the transsilencing Krueppel-associated box (KRAB) domain of the human *kox*-1 gene. Following constitutive expression by P_SV40_, PMS binds and silences a chimeric promoter P_PMS_ (pWH5, P_PMS_-SEAP-pA; P_PMS_, P_SV40_-O_PmeR2_) containing a tandem PMS-specific operator module O_PmeR2_ 3’ of a constitutive P_SV40_. In the absence of parabens PMS binds and silences P_PMS_-driven SEAP expression, while the presence of paraben derivatives results in the release of PMS from P_PMS_ and induces P_PMS_-driven SEAP expression. **(C)** Reporter protein-based metabolic integrity assay and **(D)** cell proliferation assay of PMA- and PMS-expressing cells. HEK-293 cells were co-transfected with pSEAP2-Control (0.3 μg, w/w), different amounts of pWH8 (P_SV40_-PMA-pA) or pWH9 (P_SV40_-PMS-pA) (0–0.3 μg, w/w) and optionally with the isogenic empty vector pMM15 that serves as a filler plasmid to keep the total transfected DNA constant (0.6 μg). The resulting SEAP levels (C) and viable cell numbers (D) of the transfected HEK-293 populations were profiled for up to 60 h. **(E)** Viability of human cells after exposure to different paraben derivatives. HEK-293 cells were cultivated in medium containing 0–200 μM of different paraben derivatives (MP, methylparaben; EP, ethylparaben; PP, propylparaben; BP, butylparaben; iBP, isobutylparaben) for 48 h before cell viability was scored. **(F)** Reporter protein-based metabolic integrity assay for paraben derivatives. HEK-293 cells were transfected with the constitutive SEAP expression vector pSEAP2-Control and cultivated in medium containing 0–200 μM of different parabens (MP, methylparaben; EP, ethylparaben; PP, propylparaben; BP, butylparaben; iBP, isobutylparaben) for 48 h before SEAP levels were profiled in the culture supernatant. **(G)** Dose-dependent paraben-repressible SEAP expression (PAR_OFF_ system). HEK-293 cells were co-transfected with pWH8 and pWH10 and cultivated for 48 h in medium containing 0–200 μM of different parabens (MP, methylparaben; EP, ethylparaben; PP, propylparaben) before SEAP levels were profiled in the culture supernatant. Repression factors: MP: 3; EP: 3; PP: 23. **(H)** Dose-dependent paraben-inducible SEAP expression (PAR_ON_ system). HEK-293 cells were co-transfected with pWH9 and pWH5 and cultivated for 48 h in medium containing 0–200 μM of different parabens (MP, methylparaben; EP, ethylparaben; PP, propylparaben) before SEAP levels were profiled in the culture supernatant. Induction factors: MP: 48; EP: 106; PP: 175. All data are shown as the mean ± SD, *n* = 3 independent experiments.

PAR_OFF_ consists of the synthetic mammalian transcription factor PMA (paraben-mediated transactivator; pWH8, P_SV40_-PMA-pA; PMA, PmeR-VP16), engineered by fusing PmeR's C-terminus to the *Herpes simplex* virus-derived transactivation domain (VP16), that modulates the activity of synthetic P_PMA_ promoters (pWH10, P_PMA_-SEAP-pA; P_PMA_, O_PmeR2_-P_hCMVmin_), containing the PMA-specific tandem operator module O_PmeR2_ 5’ of a minimal version of the human cytomegalovirus immediate-early promoter (P_hCMVmin_), in a paraben-responsive manner: In the absence of parabens, PMA binds and activates P_PMA_-driven transgene expression while paraben derivatives prevent the PMA–P_PMA_ interaction and repress target-gene expression (Figure 1A).

PAR_ON_ consists of a synthetic mammalian transcription silencer PMS (paraben-mediated transsilencer; pWH9, P_SV40_-PMS-pA; PMS, PmeR-KRAB), engineered by fusing PmeR's C-terminus to the Krueppel-associated box (KRAB) domain of the human *kox*-1 gene, that modulates the activity of synthetic P_PMS_ promoters (pWH5, P_PMS_-SEAP-pA; P_PMS_, P_SV40_-O_PmeR2_), containing the PMS-specific tandem operator module O_PmeR2_ 3’ of the constitutive simian virus 40 promoter (P_SV40_), in a paraben-responsive manner. In the absence of parabens PMS binds and represses P_PMS_ while paraben derivatives prevent the PMS-P_PMS_ interaction and induce target-gene expression (Figure 1B).

To assess the impact of PMA and PMS expression on the metabolic integrity and the viability of mammalian cells, we co-transfected HEK-293 with pSEAP2-Control and increasing concentrations of either the PMA-encoding vector pWH8 (P_SV40_-PMA-pA) or the PMS-encoding vector pWH9 (P_SV40_-PMA-pA) and profiled SEAP production (Figure 1C) as well as the viable cell number of the cell population for up to 60 h (Figure 1D). Likewise, to assess the impact of parabens on the viability and the metabolic integrity of mammalian cells, we cultivated HEK-293 cells in medium containing different concentrations (0–200 μM) of MP, EP PP, BP and iBP, which are most commonly used as cosmetics additives, and profiled the percent viable cell number (Figure 1E) and the overall SEAP production capacity of treated cell lines (Figure 1F). Collectively, these results show that none of the parabens impaired cell viability and that only parabens with longer alkyl chains such as BP and iBP decreased constitutive SEAP expression, suggesting that they have a negative impact on the cell physiology within the tested concentration range (0–200 μM). However, neither MP, EP nor PP did impair the SEAP production capacity of HEK-293 (Figure 1F). When using increasing concentrations (0–200 μM) of MP, EP and PP to control SEAP expression in PAR_OFF_- (pWH8/pWH10) or PAR_ON_- (pWH9/pWH5)-engineered HEK-293 cells product gene expression was repressed (Figure 1G) or induced (Figure 1H) in a dose-dependent manner, respectively. Since PP showed the tightest repression and highest induction for PAR_OFF_ as well as PAR_ON_, this paraben derivative was used in all follow-up experiments.

### Validation of PAR_OFF_- and PAR_ON_-controlled transgene expression in different mammalian cell lines

**Figure 2.**
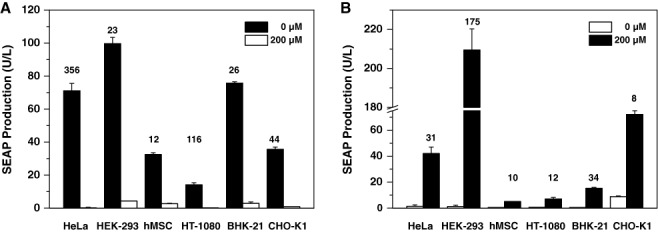
**(A)** Validation of the PAR_OFF_ system in different mammalian cell lines. HeLa, HEK-293, hMSC-TERT, HT-1080, BHK-21 and CHO-K1 were co-transfected with pWH8 and pWH10 and cultivated for 48 h in the presence (200 μM) or absence (0 μM) of propylparaben before SEAP levels were profiled in the culture supernatant. Values above the cell line-specific SEAP expression bars indicate the fold repression factor. **(B)** Validation of the PAR_ON_ system in different mammalian cell lines. HeLa, HEK-293, HT-1080, hMSC-TERT, BHK-21 and CHO-K1 were co-transfected with pWH9 and pWH5 and cultivated for 48 h in the presence (200 μM) or absence (0 μM) of propylparaben before SEAP levels were profiled in the culture supernatant. Values above the cell line-specific SEAP expression bars indicate the fold induction factor. All data are shown as the mean ± SD, *n* = 3 independent experiments.

To assess the potential of the paraben-responsive transgene expression devices for a wide range of applications, we tested the performance of the PAR_OFF_ and PAR_ON_ systems in different cell types. Therefore, the PAR_OFF_ system (pWH8/pWH10) was transfected into four human (HEK-293, HeLa, HT-1080, hMSC-TERT) and two animal (CHO-K1, BHK-21) cell lines and cultivated for 48 h in the presence and absence of 200 μM PP before SEAP was profiled in the culture supernatants. The PAR_OFF_ system was functional in all tested cell lines and showed the best regulation performance in HeLa and HEK-293 cells, combining highest absolute SEAP expression levels (HeLa: 71.1 ± 4.4 U/L; HEK-293: 99.6 ± 3.8 U/L) in the PP-free induced state with almost complete PP-mediated repression (HeLa: 0.2 ± 0.3 U/L; HEK-293: 4.3 ± 0.1 U/L) (Figure 2A).

Likewise, when cultivating the same set of mammalian cell lines engineered with the PAR_ON_ system (pWH9/pWH5) for 48 h in the presence and absence of 200 μM PP, resulting SEAP expression levels in the supernatant indicated that the PAR_ON_ system too was functional in a wide variety of cell types. However, the PAR_ON_ gene switch showed the optimal PP-inducible expression control in HEK-293 cells with minimal SEAP expression (1.2 ± 1.0 U/L) in the absence and high maximum expression induction (210.2 ± 10.9 U/L) in the presence of the trigger compound (Figure 2B).

Collectively, both ON-type and OFF-type PP-controlled transgene expression systems are functional in different mammalian cell types from various species, suggesting that these control devices will have broad utility for a wide range of applications.

### Construction, selection and characterization of stably transgenic paraben-regulated mammalian cell lines

**Figure 3.**
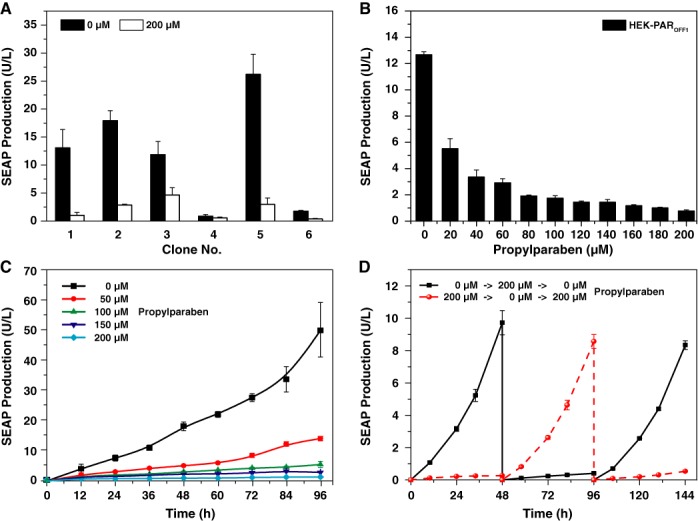

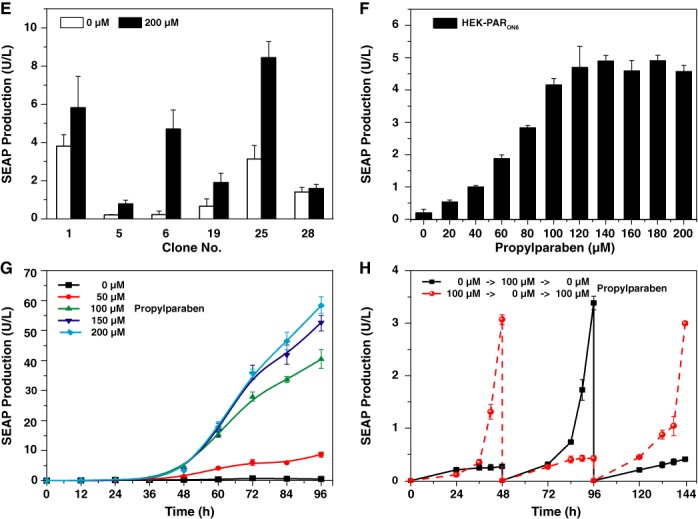
Design and characterization of clonal PAR_OFF_- and PAR_ON_-transgenic cell lines. **(A)** Propylparaben-repressible SEAP expression of different PAR_OFF_-transgenic cell clones (HEK-PAR_OFF_). HEK-293 cells were stably co-transfected with pWH8 (P_SV40_-PMA-pA) and pWH10 (P_PMA_-SEAP-pA) and six randomly selected cell clones were profiled for their propylparaben-repressible SEAP regulation performance by cultivating them for 48 h in the presence (200 μM) and absence (0 μM) of propylparaben. **(B)** Dose-dependent SEAP expression profile of the HEK-PAR_OFF1_ cell line after cultivation for 48 h in the presence of increasing propylparaben concentrations (0–200 μM). **(C)** SEAP expression kinetics of HEK-PAR_OFF1_ (2×10^5^ cells/ml) cultivated for 96 h in culture medium containing increasing concentrations of propylparaben (0–200 μM). Prior to addition of propylparaben (time point 0), HEK-PAR_OFF1_ cells were cultivated in paraben-free medium. **(D)** Reversibility of HEK-PAR_OFF1_-mediated SEAP expression. 2×10^5^ cells/ml HEK-PAR_OFF1_ cells were cultivated for 144 h while alternating the propylparaben concentrations from 0 to 200 μM and adjusting the cell density to 2×10^5^ every 48 h. **(E)** Propylparaben-inducible SEAP expression of different PAR_ON_-transgenic cell clones (HEK-PAR_ON_). HEK-293 cells were stably co-transfected with pWH9 (P_SV40_-PMS-pA) and pWH5 (P_PMS_-SEAP-pA) and six randomly selected cell clones were profiled for their propylparaben-inducible SEAP regulation performance by cultivating them for 48 h in the presence (200 μM) and absence (0 μM) of propylparaben. **(F)** Dose-dependent SEAP expression profile of the HEK-PAR_ON6_ cell line after cultivation for 48 h in the presence of increasing propylparaben concentrations (0–200 μM). **(G)** SEAP expression kinetics of HEK-PAR_ON6_ (2×10^5^ cells/ml) cultivated for 96 h in culture medium containing increasing concentrations of propylparaben (0–200 μM). Prior to addition of propylparaben (time point 0), HEK-PAR_ON6_ cells were cultivated in paraben-free medium. **(H)** Reversibility of HEK-PAR_ON6_-mediated SEAP expression. 2×10^5^ cells/ml HEK-PAR_ON6_ cells were cultivated for 144 h while alternating the concentrations from 0 to 100 μM and adjusting the cell density to 2×10^5^ every 48 h. All data are shown as the mean ± SD, *n* = 3 independent experiments.

We have generated six clonal double-transgenic cell lines (HEK-PAR_OFF1–6_), in which SEAP is controlled by the PAR_OFF_ system, by stably co-transfecting pWH8 and pWH10 into HEK-293. All of these transgenic HEK-PAR_OFF_ cell lines showed PP-repressible regulation profiles, but differed in their overall SEAP expression performance characterized by specific maximum and basal transgene expression signatures (Figure 3A). Due to the combination of the highest induction ratio and the lowest IC_50_-concentration of PP (Figure 3B), HEK-PAR_OFF1_ emerged as the best-in-class transgenic cell line that was chosen for all follow-up studies. Also, HEK-PAR_OFF1_ showed dose-dependent SEAP repression (Figure 3B), dose-dependent SEAP induction kinetics (Figure 3C) and completely reversible SEAP expression profiles when alternating the presence and absence of PP in the culture medium (Figure 3D).

Likewise, we have generated six clonal double-transgenic cell lines, in which SEAP is controlled by the PAR_ON_ system, by stably co-transfecting pWH9 and pWH5 into HEK-293. All of these transgenic HEK-PAR_ON_ cell lines showed PP-inducible regulation profiles, but differed in their overall SEAP induction ratio (Figure 3E). The cell clone HEK-PAR_ON6_ showed (i) a near perfect induction ratio characterized by almost undetectable basal expression in the absence of PP and high maximum expression levels in the presence of PP (Figure 3F), (ii) robust PP dose-dependent SEAP production kinetics (Figure 3G) and completely reversible SEAP expression kinetics when alternating presence and absence of the trigger compound in the culture medium (Figure 3H).

Because of the integration of the transgene expression units into random chromosomal loci by illegitimate recombination, gene switch performance is dependent on the chromosomal context and can therefore dramatically vary among different stable cell clones (Figure 3A and E) (45,46).

### Comparative performance analysis of the PAR_ON_ and PEACE_ON_ systems

**Figure 4.**
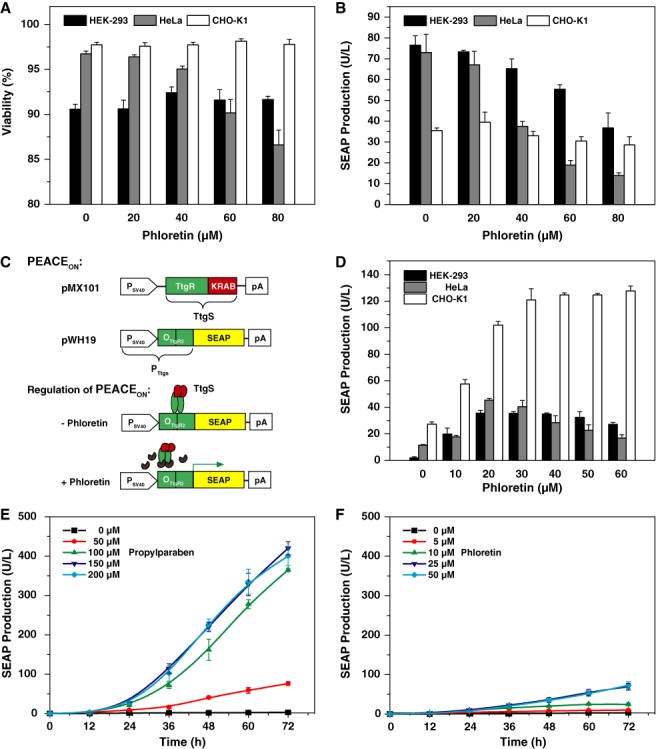
Comparative performance analysis of PAR_ON_ and PEACE_ON_ gene switches. **(A)** Viability of mammalian cells after exposure to different concentrations of phloretin. HEK-293, HeLa and CHO-K1 were cultivated in cell culture medium containing different concentrations of phloretin (0–80 μM) for 48 h before cell viability was scored. **(B)** Reporter protein-based metabolic integrity assay for phloretin. HEK-293, HeLa and CHO-K1 cells were transfected with the constitutive SEAP expression vector pSEAP2-Control and cultivated in cell culture medium containing different amounts of phloretin (0–80 μM) for 48 h before SEAP levels were profiled in the culture supernatant. **(C)** Design and functionality of a phloretin-inducible transcription-control system PEACE_ON_. The transsilencer (pMX101, P_SV40_-TtgS-pA; TtgS, TtgR-KRAB) and the reporter plasmids (pWH19, P_SV40_-O_TtgR2_-SEAP-pA) of the PEACE_ON_ system are isogenic to the plasmids pWH9 and pWH5 of the PAR_ON_ system, respectively. Following constitutive expression by the Simian virus 40 promoter (P_SV40_), TtgS binds and silences a chimeric promoter P_TtgS_ (pWH19, P_TtgS_-SEAP-pA; P_TtgS_, P_SV40_-O_TtgR2_) containing a tandem TtgS-specific operator module O_TtgR2_ 3’ of a constitutive P_SV40_. In the absence of phloretin TtgS binds and silences P_TtgS_-driven SEAP expression, while the presence of phloretin results in the release of TtgS from P_TtgS_ and induces P_TtgS_-driven SEAP expression. **(D)** Dose-dependent phloretin-inducible SEAP expression (PEACE_ON_). HEK-293, HeLa and CHO-K1 cells were co-transfected with the PEACE_ON_ control components (pMX101/pWH19) and cultivated for 48 h in the presence of increasing phloretin concentrations (0–60 μM) before SEAP levels were profiled in the culture supernatants. **(E, F)** Control kinetics of the (E) PAR_ON_ and (F) PEACE_ON_ systems. HEK-293 cells were co-transfected with the PAR_ON_ (pWH9/pWH5) or PEACE_ON_- (pMX101/pWH19) control components and cultivated for 72 h in the presence of increasing concentrations of the corresponding trigger compounds propylparaben (0–200 μM) or phloretin (0–50 μM) before SEAP levels were profiled in the culture supernatants. All data are shown as the mean ± SD, *n* = 3 independent experiments.

ON-type gene control systems that induce target-gene expression in response to a transient molecular cue is the preferred gene switch design, because the trigger compound only needs to be administered upon induction. In contrast, OFF-type switches require continuous presence of control compounds for repression and active removal for induction, which limits applications of this control topology *in vivo*. However, ON-type switches are more challenging to design, as they have to be extremely tight so that the target protein does not accumulate to significant levels even in the absence of the trigger compound. We have therefore redesigned the phloretin-adjustable control element (PEACE), the pioneering OFF-type design that enabled transdermal control by the apple metabolite phloretin (12), into an isogenic ON-type design (pMX101, P_SV40_-TtgR-KRAB-pA; pWH19, P_SV40_-O_TtgR2_-SEAP-pA) for comparative performance analysis with the PAR_ON_ system (pWH9/pWH5) (Figure 4). To assess the impact of phloretin on the viability and the metabolic integrity of mammalian cells, we performed the same reporter protein-based assay as for parabens (Figure 1E and F) and found that although phloretin did not impair the viable fraction of treated cells within the standard PEACE-inducing concentration range (0–50 μM, (12)) (Figure 4A), higher concentrations decreased constitutive SEAP production capacity of mammalian cells (Figure 4B). Therefore, the phloretin-inducible transgene expression switch (Figure 4C) may not reach optimal peak expression levels in various human cell lines. Also, the induction factor reached by the PEACE_ON_ system was lower in all tested mammalian cell lines (Figure 4D) when compared to the PAR_ON_ system (Figure 2B). Moreover, the PP-controlled transgene expression system delivers precisely adjustable induction kinetics (Figure 4E) and shows improved dose-dependent transgene-induction characteristics compared to its phloretin-regulated counterpart (Figure 4F). Collectively, these data suggest that the PAR_ON_ gene expression system will be the preferred control design for percutaneous control of transgene expression.

### Cosmetics-controlled transgene expression

**Figure 5.**
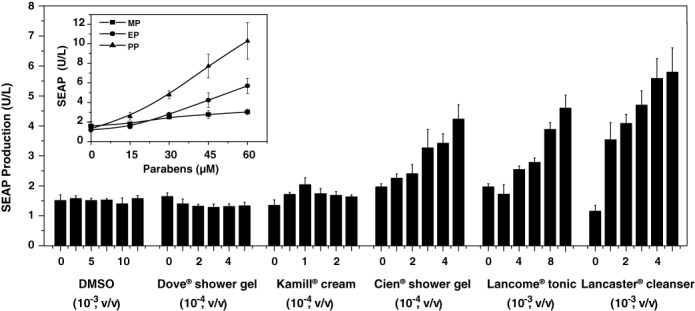
Cosmetics-induced PAR_ON_-dependent SEAP expression *in vitro*. HeLa cells were co-transfected with pWH9 and pWH5 and cultivated in cell culture medium containing different cosmetic products (Dove^®^ shower gel, Kamill^®^ hand-cream, Lancôme Paris^®^ Tonic, Cien^®^ shower gel, Lancaster^®^ Express Cleanser) at different dilutions (v/v) or different concentrations (0–60 μM) of various parabens (MP, methylparaben; EP, ethylparaben; PP, propylparaben) (insert). Addition of the DMSO was used as solvent control. Forty-eight hours after addition of cosmetics, SEAP levels in the culture supernatant were profiled. All data are shown as the mean ± SD, *n* = 3 independent experiments.

In order to evaluate whether the paraben levels in cosmetics approved by the Personal Care Products Council (<0.8%, (32)) matches the sensitivity range of the PAR_ON_ system, we exposed pWH9/pWH5-transgenic HeLa cultures to different amounts of commercial skin-care products including toner solutions (cleanser (Lancaster^®^), skin toner (Lancôme^®^)), emulsion creams (hand cream (Kamill^®^) and shower gels (Dove^®^, Cien^®^)) (Figure 5). Although all of the skin-care products had to be diluted to reduce the cytotoxicity of the soap components, all paraben-containing products were able to dose-dependently induce PAR_ON_-driven SEAP expression. Dove^®^ shower gel, which was explicitly declared as paraben-free, was indeed not inducing the PAR_ON_ device and served as a negative control (Figure 5). The results indicated that the PAR_ON_ system could be regulated by paraben-containing skin-care and hygiene products *in vitro* with Lancaster^®^ cleanser and Cien^®^ shower gel showing the best dose-dependent induction performance among all tested toner solutions and emulsions, respectively (Figure 5). However, since the induction performance of the cosmetics could be confounded by the cytotoxicity of its soap components for cells grown in culture the true paraben-based control capacity of the cosmetics can only be assessed by percutaneous control of SC PAR_ON_-transgenic designer cell implants in an animal model.

### Percutaneous control of SC implants by topical administration of cosmetic skin-care products

**Figure 6.**
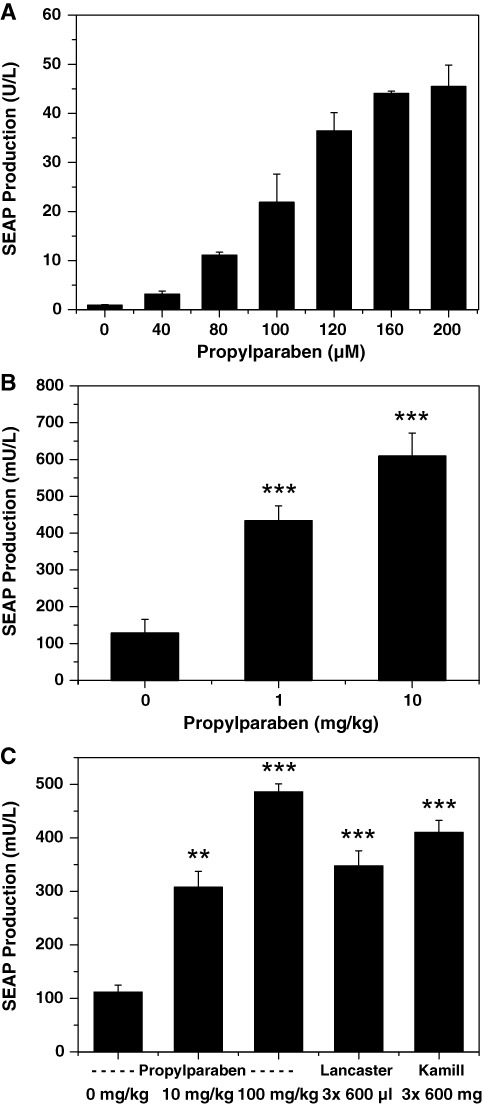
Validation of the PAR_ON_-system in mice. **(A)** SEAP induction profiles of microencapsulated PAR_ON_-transgenic HEK-293 cells *in culture*. pWH9/pWH5-transgenic microencapsulated HEK-293 cells (1×10^5^ cells, 500 capsules, 200 cells/capsule) were cultivated in 500 μl cell culture medium containing different concentrations of propylparaben (0–200 μM) for 48 h before SEAP levels were profiled in the culture supernatant. The data are shown as the mean ± SD, *n* = 3. **(B)** Dose-dependent propylparaben-induced SEAP expression in mice. The same batch of microencapsulated pWH9/pWH5-transgenic HEK-293 cells (2×10^6^ cells, 10 000 capsules, 200 cells/capsule) was intraperitoneally implanted into mice, which received different daily injections of propylparaben (0–10 mg/kg) for 48 h before SEAP levels were quantified in their bloodstream. **(C)** Percutaneous control of transgene expression in SC implants by topical application of paraben-containing cosmetics. The same batch of microencapsulated pWH9/pWH5-transgenic HEK-293 cells (2×10^6^ cells, 10 000 capsules, 200 cells/capsule) was subcutaneously implanted into mice, which received thrice daily topical applications of paraben-containing cosmetics (three daily administrations: 3×600 μg Kamill^®^ hand cream; 3×600 μl Lancaster^®^ cleanser). Solutions containing different concentrations of propylparaben (three daily administrations: 3×50 μl for a total of 0, 10, 100 mg/kg day^−1^) were used as controls. The SEAP levels in the bloodstream of treated animals were profiled after 48 h. The data are shown as the mean ± SEM, statistics by two-tailed t test, *n* = 8 mice. **P < 0.01, ***P < 0.001 versus control.

To assess the performance of the PAR_ON_ system *in vivo*, we microencapsulated pWH9/pWH5-transgenic HEK-293 cells into coherent, semi-permeable (allowing free diffusion of nutrients, waste metabolites and SEAP) and immunoprotective (pore-size tuned to prevent transfer of immunoglobulins) beads made of alginate-poly-(L-lysine)-alginate, a clinically licensed material that was shown to enable vascularization and connection of entrapped designer cells to the bloodstream (47,48) and has been successfully tested in human-clinical trials (49). Paraben-inducible SEAP expression of microencapsulated PAR_ON_-transgenic designer cells was validated in cell culture (Figure 6A) before the same batch was either intraperitoneally (Figure 6B) or subcutaneously implanted into mice (Figure 6C). Animals treated with IP implants received one-dose-per-day of three different concentrations of PP (0–10 mg/kg). Analysis of blood-paraben levels confirmed regulation-effective paraben concentrations in circulation for up to 24 h (60.6 ± 9.1 μM), corroborating established paraben pharmacokinetics (50,51). Forty-eight hours after paraben administration, SEAP expression was profiled in the bloodstream of treated mice (Figure 6B). This data set confirmed dose-dependent high-level performance of the preferred PAR_ON_ gene switch *in vivo* yet did not reveal whether the device was sufficiently sensitive to accept percutaneous control input or whether parabens contained in commercial skin-care products would cross the skin to program transgene expression in SC designer cell implants. Therefore, we treated mice with SC PAR_ON_-transgenic designer cell implants with three topical applications per day of undiluted Lancaster^®^ cleanser or Kamill^®^ hand cream or solutions containing three different concentrations of PP (0–100 mg/kg) as controls (Figure 6C). Collectively, this data show that paraben (i) is able to cross the skin, (ii) can remote control cellular behavior inside the body in a non-invasive manner simply by topical application of paraben and that (iii) commercial skin-care and hygiene products contain sufficient paraben to program designer cell implants using a typical three-times-per-day application frequency.

## DISCUSSION

With the ambition to use orthogonal gene switches for the control of therapeutic transgene expression dosing in future gene- and cell-based therapies, the quest for the ideal inducer compounds has just started. While clinically licensed drugs such as antibiotics (13,52–53), hormones (54) and antidiabetics (55) have secondary therapeutic effects and collateral side effects, amino acids (56,57), vitamins (44,58) and metabolites (59) are non-orthogonal and require control concentrations that permanently exceed physiologic levels, food components and food additives such as phloretin (12), preservatives (60) and flavors (60,61) that limit the choice of diet and traceless inducers such as temperature (62,63), light (4) and radiowaves (64) are ubiquitous environmental cues that are impossible to avoid and use for exclusive therapeutic control.

Besides the type of the trigger compound, the administration route will be of prime importance for dosing and patient compliance. In contrast to conventional compound injections, which require medical care, and oral administration, which is limited by the hepatic first-pass effect, transdermal delivery of trigger compounds would improve patient compliance, enable local administration and eliminate the hepatic first-pass effect as well as the need for assistance by trained medical personnel. Pioneering efforts to establish transdermal gene expression control culminated in the design of the phloretin-repressible control element (PEACE), whose expression could be fine-tuned in SC implants by topical application of the penetration enhancer phloretin (12). However, since the PEACE system shows lower trigger sensitivity compared to the paraben control switch *in vivo*, phloretin needs to be administered at much higher concentrations (1680 mg/kg, (12); compared to 100 mg/kg for PP), which are not present at control-effective levels in off-the-shelf cosmetics.

Collectively, an ideal control compound for therapeutic transgene control should be (i) physiologically inert to prevent any metabolic crosstalk, (ii) clinically licensed to guarantee safety, (iii) rapidly cleared from peripheral circulation to support reversibility of transgene expression and (iv) enable percutaneous control following topical administration.

With their validated generic design principle, their high-performance ON-/OFF-type switch dynamics, combining adjustability and reversibly with low basal as well as high maximum expression profiles, and their responsiveness to the physiologically inert cosmetics preservative PP, the PAR_OFF_ and PAR_ON_ devices meet with all criteria of an ideal gene switch for therapeutic transgene control at a high standard (65). In direct comparison with the ON-type PEACE system providing phloretin-modulated expression control, the isogenic PAR_ON_ gene switch shows higher induction factors and faster induction kinetics in the tested mammalian cell line. Despite the long history of commonplace application as cosmetics preservative, the finding of increased paraben levels in breast cancer tissue has triggered discussions about the role of parabens in the development of breast cancer (66–68). Although the causal connection has not been scientifically proven and authorities have left the NOAEL (1000 mg/kg day^−1^) unchanged, an increasing number of consumer product companies feel the consumer pressure and produce paraben-free skin-care products. The PAR_ON_ system is sufficiently sensitive to reliably test the presence of parabens in cosmetics. For example, using the PAR_ON_ system we could confirm the presence of parabens in Kamill^®^ hand cream, Lancaster^®^ cleanser and Lancôme Paris^®^ toner as well as the absence of regulation-effective paraben levels in the Dove^®^ shower gel. The paraben-inducible gene switch may therefore also be used to augment animal models for toxicology studies. Furthermore, using paraben-spiked paraben-free commercial skin-care products would allow orthogonal percutaneous remote control of transgenes for therapeutic purposes without interfering with the patients’ lifestyle or hygiene habits. Because of the duty of declaration for parabens in consumer care products and cosmetics, the risk of accidental exposure of individuals to parabens is considered negligible.

Collectively, due to the combination of high skin permeation capacity, low toxicity and a regulation-effective concentration range that fully matches FDA-approved doses (<0.1%), parabens particularly qualify for remote control of therapeutic transgene expression in SC designer cell implants for safe transdermal therapeutic applications in the future.
